# Clinical validation of terminological subsets of the international classification for nursing practice: a scoping review

**DOI:** 10.1590/0034-7167-2024-0203

**Published:** 2025-04-28

**Authors:** Fernanda de Freitas Ferreira, Silvia Maria de Sá Basílio Lins, Harlon França de Menezes, Rebecca Stefany da Costa Santos, Juliana Otaciana dos Santos, Richardson Augusto Rosendo da Silva

**Affiliations:** I Universidade Federal Fluminense. Niterói, Rio de Janeiro, Brazil; II Universidade Federal do Rio Grande do Norte. Natal, Rio Grande do Norte, Brazil

**Keywords:** Nursing Process, Validation Study, Standardized Nursing Terminology, Nursing Diagnosis, Clinical Nursing Research, Proceso de Enfermería, Estudio de Validación, Terminología Normalizada de Enfermería, Diagnóstico de Enfermería, Investigación en Enfermería Clínica

## Abstract

**Objectives:**

to map the scientific evidence on the clinical validation of terminological subsets of the International Classification for Nursing Practice (ICNP^®^).

**Methods:**

a scoping review was conducted between January and February 2024 across nine databases. The review adhered to the guidelines established by the JBI and followed the checklist for the Preferred Reporting Items for Systematic Reviews and Meta-Analyses Extension for Scoping Reviews. Data analysis was performed using simple descriptive statistics and lexical analysis with IRaMuTeQ software.

**Results:**

fifteen studies were selected, with the majority conducted in Brazil (93.3%). Seven were master’s dissertations (46.7%); 86.7% focused on adult and elderly health, while 13.3% addressed maternal and child health. Eight studies were conducted at the Federal University of Paraíba (53.3%), and ten works adopted case study designs (66.7%).

**Conclusions:**

there is a lack of progress, as the studies are in their early stages and require greater methodological consistency and broader availability.

## RESUMEN

**Objetivos:**

mapear las evidencias científicas sobre la validación clínica de subconjuntos terminológicos de la Clasificación Internacional para la Práctica de Enfermería (CIPE^®^).

**Métodos:**

revisión de alcance realizada entre enero y febrero de 2024 en nueve bases de datos. La elaboración siguió las directrices establecidas por el JBI, así como el listado de verificación *Preferred Reporting Items for Systematic Reviews and Meta-Analyses Extension for Scoping Reviews*. El análisis de los datos se llevó a cabo mediante estadística descriptiva simple y análisis léxico utilizando el software IRaMuTeQ.

**Resultados:**

se seleccionaron 15 estudios, de los cuales la mayoría fueron realizados en Brasil (93,3%); siete fueron estudios de disertación (46,7%); el 86,7% se enfocaron en la salud del adulto y del anciano, y el 13,3% se referían a la salud maternoinfantil. Ocho estudios se llevaron a cabo en la Universidad Federal de Paraíba (53,3%) y diez producciones adoptaron estudios de caso (66,7%).

**Conclusiones:**

se observa una carencia de avances, ya que los estudios son incipientes y requieren mayor disponibilidad y uniformidad en los métodos.

## INTRODUCTION

The construction of the Nursing Process (NP) relies on the use of standardized language systems in clinical practice, which contribute to clinical reasoning and decision-making aimed at improving patient health and human responses. In this context, the significance of clinical indicators for nursing diagnoses becomes evident, as they provide essential evidence for identifying and applying interventions. Accurate and validated nursing diagnoses guide the selection of appropriate interventions capable of producing desired outcomes, thereby supporting research, management, education, and the development of public policy proposals^(1,2)^.

Research indicates that the adoption of a standardized language in Nursing aims to consolidate this profession as a science, establishing a systematic care that is based on evidence and guided by the health priorities relevant to its field of activity. Only through a specific and uniform terminology will nursing professionals be able to demonstrate their scientific role in the scope of health care, using this language in the various phases of the NP^(3,4)^.

With the publication of the recent Resolution of the Federal Nursing Council 736 of 2024, it is clear that standardized language systems support the NP, based on the best levels of scientific evidence. Language systems have concepts to be created, studied and validated, which is why the validation process is essential to improve and authenticate them, with a view to developing dynamic knowledge and investigations that involve new tools with the possibility of applying it in the daily life of the professional who produces it^(5,6)^.

Different types and methods of validation are applied in this field, including content, concept, semantic, construct, criterion, and clinical validation. Among these, clinical validation has been further developed and utilized as a methodological process in research. Therefore, it is urgent to discuss the key aspects of its development, as it represents a crucial stage in constructing such studies and underscores the need for refinement^(7)^.

The methodological guidelines from the International Council of Nurses (ICN) emphasize that nursing diagnoses, outcomes, and intervention statements, organized in the form of Terminological Subsets of the International Classification for Nursing Practice (ICNP^®^), must undergo clinical validation. This validation should be conducted based on the phases of the NP: assessment, diagnosis, planning, implementation, and evaluation^(1)^.

However, the Brazilian method does not include a clinical validation phase for the subset, although it acknowledges its importance and encourages studies aimed at clinically validating the constructed subset. As a result, studies of this nature are generally linked to diagnostic concepts, outcomes, or nursing interventions, rather than a terminological subset. This creates a knowledge gap that needs to be addressed by researchers in the field^(1)^.

To better understand what has been documented about clinical validation and its impact on ICNP^®^ terminological subsets, this study was designed as a guiding tool for researchers seeking to refine and validate data from their practice, offering support for disseminating and producing knowledge.

## OBJECTIVES

To map the scientific evidence on the clinical validation of ICNP^®^ terminological subsets.

## METHODS

### Ethical Considerations

As this is a review study, ethical approval was not required, since the material used is in the public domain and does not involve human participants.

### Study Design

This analysis constitutes a scoping review, organized in accordance with the guidelines of the international *Preferred Reporting Items for Systematic Reviews and Meta-Analyses* (PRISMA)^(8)^ and the method suggested by the JBI^(9)^. Such reviews primarily aim to provide an overview of concepts that underpin a specific field of knowledge, assess the depth, breadth, and nature of the research conducted, compile and disseminate the data obtained, and identify existing gaps in the research^(9)^. The protocol was registered with the Open Science Framework (OSF) and is available at (https://doi.org/10.17605/OSF.IO/DWYKV).

### Search Strategy and Data Sources

To fulfill the stages of this study, the study’s objective and research question were defined using the mnemonic combination PCC: P (Population) - Articles published on terminological subsets; C (Concept) - Clinical validation methods; C (Context) - Studies conducted in any country and type of institution. Based on these definitions, the study objective and the following guiding question were established: What methods are used for the clinical validation of ICNP^®^ terminological subsets in the global literature?

In developing the search strategy, controlled descriptors from *Medical Subject Headings* (MeSH) and *Descriptors in Health Sciences* (DeCS), as well as keywords, were used to expand the available literature. Boolean operators “OR” and “AND” were also employed, along with the selection of synonyms found in MeSH and DeCS. The search strategy formulation was carried out in collaboration with a librarian from the Regional Nursing Council of Minas Gerais (COREN-MG). The DeCS/MeSH terms used were: Standardized Nursing Terminology and ICNP^®^, applying the boolean operator “OR”: “Standardized Nursing Terminology” OR “*Terminologia Padronizada em Enfermagem*” OR “*Terminología Normalizada de Enfermería*” OR “CIPE^®^” OR “ICNP^®^” (Chart 1).

**Chart 1:** Search Strategy, Niterói, Rio de Janeiro, Brazil, 2024

Data Sources	Search Strategy
BVS	*(“Terminologia Padronizada em Enfermagem”* OR “Standardized Nursing Terminology”) OR *“Terminología Normalizada de Enfermería”* OR CIPE OR ICNP*; “*Estudos de Validação como Assunto*”* OR *“Validation Studies as Topic”* OR *“Estudios de Validación como Asunto”* OR *“Estudo de Validação”* OR *“*Validation Study*”* OR *“Estudio de Validación”*
PUBMED	“Standardized Nursing Terminology” OR “ICNP Terminology” OR “Validation Studies as Topic” OR “Validation Study” OR Validation

*BVS - Virtual Health Library.*

The investigation was conducted between January and February 2024, covering the following databases, repositories, and directories: MEDLINE (via PubMed) and CINAHL (through the EBSCO platform). Subsequently, a broader search was conducted using the same keywords and search criteria across the Web of Science, Science Direct (via the Scopus platform), Cochrane Database of Systematic Reviews, as well as the Virtual Health Library (BVS) portal, the Brazilian Digital Library of Theses and Dissertations (BDTD), the Brazilian Institute of Information in Science and Technology (IBICT), and the Science.gov platform for exploring gray literature.

### Data Collection, Organization, and Analysis

Complete studies were considered for the research based on a title review conducted by two independent authors. Following the reading of abstracts, a preliminary selection was made of studies that met the inclusion criteria for this review. In cases of doubt or disagreement regarding the analysis of titles, abstracts, and study relevance, the study supervisor was consulted. Rayyan^®^ software was used to verify duplicate articles and to add explanatory labels and justifications for inclusion or exclusion.

The refinement of studies was based on established eligibility criteria. Studies related to ICNP and validation, available in full and free of charge, were included. Studies that did not address clinical validation methods were excluded. No temporal or linguistic restrictions were applied to ensure the full scope of studies was covered.

Data were extracted, organized, and categorized in Microsoft Office Excel^®^ spreadsheets in the following order: database, year of publication, location of publication, theoretical/conceptual framework, sample, follow-up, data collection technique, analysis, level of evidence, degree of recommendation, and theoretical support. The level of evidence (LE) and degree of recommendation were determined using the prerogatives of the Oxford Centre for Evidence-Based Medicine^(10)^.

Subsequently, data were analyzed in two ways: 1) using descriptive statistics (relative and absolute), presented in tables; and 2) through lexical analysis performed using the software *Interface de R pour les Analyse Multidimensionnelles de Textes et de Questionnaires* (IRaMuTeQ), version 0.7 Alpha 2, and R version 3.2.3, which provides a quantitative approach to qualitative data^(11)^.

To build the textual corpus, study abstracts were included, from which Text Segments (TS) and the grouping of the most significant words were identified^(11)^. Among the analysis methods allowed by the software, the results of this study were presented through the following analyses: 1) Word Cloud, which graphically represents the occurrences within the textual corpus, indicating that each word’s size is proportional to its frequency, displayed in a figure that does not provide descriptive data but allows for the rapid identification of the most significant terms within the text corpus^(12)^; and 2) Similarity Tree, which is created based on word occurrences present in the text segments^(13)^. This analysis visually demonstrates the origin and connection of words. Both analyses were discussed with the support of the literature.

## RESULTS

A total of 2,953 studies were identified in the databases, of which 1,551 were removed due to duplication. This left 1,402 studies. After reviewing the titles and abstracts, 1,273 were excluded for not meeting the inclusion criteria. A total of 129 articles were selected for full-text reading, resulting in the exclusion of 85 texts that did not address ICNP terminological subsets. A second round of full-text reading was conducted with 44 articles, culminating in the exclusion of 29 studies that did not address clinical validation. Therefore, 15 studies remained, forming the focus of this scoping review.

**Figure 1:**
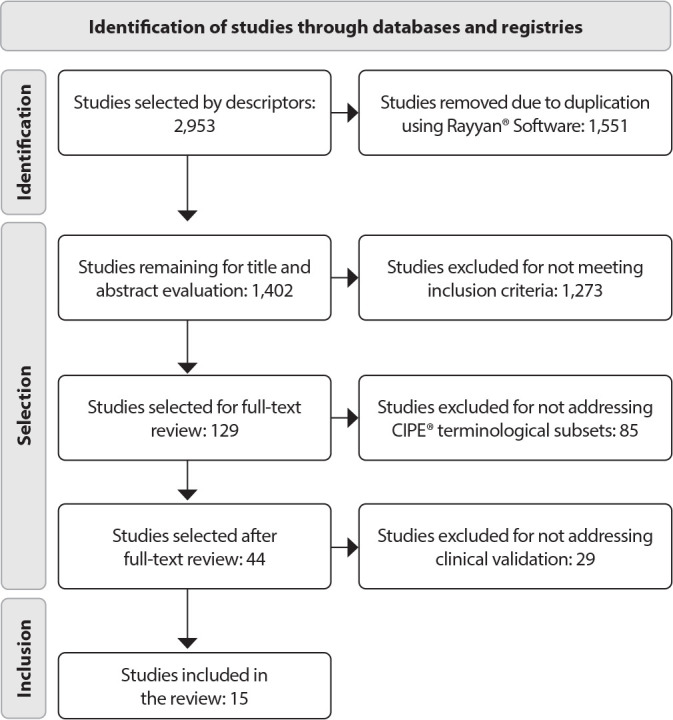
PRISMA Diagram for the Study Selection Process, 2023

Figure 1 illustrates the results of the analysis stages, following the PRISMA Flow Diagram model. The analysis of publications regarding year of publication, database, country, institution, title, and type of production was synthesized in Chart 2. The studies were numbered and ordered by ascending publication date, with the letter (E) representing “study,” followed by the corresponding number. In terms of publication year, the first study was published in 2014, with a progressive increase beginning in 2016. Brazil produced the majority of clinical validation studies (93.3%).

**Chart 2:** Characterization of studies according to code E1 to E14, year, database, country and institution of publication, title, and type of production related to the study theme, Niterói, Rio de Janeiro, Brazil, 2024

ID	Year/ Database	Country/Institution	Title	Type of Production
E1^(14)^	2014/BVS	Brazil/Federal University of Paraíba	*Validação do subconjunto terminológico da CIPE^®^ para a pessoa idosa*	Doctoral Thesis
E2^(15)^	2016/BVS	Brazil/Federal University of Paraíba	*Validação do Subconjunto Terminológico CIPE^®^ para pacientes com dor oncológica*	Doctoral Thesis
E3^(16)^	2016/BVS	Brazil/Federal University of Paraíba	*Validação da nomenclatura de diagnósticos, resultados e intervenções de enfermagem para a clínica pediátrica do Hospital Universitário da UFPB*	Master’s Dissertation
E4^(17)^	2017/BVS	Brazil/University of Brasília	*Subconjunto terminológico CIPE^®^ para a prática de enfermagem ambiental e ocupacional*	Doctoral Thesis
E5^(18)^	2017/BVS	Brazil/Federal University of Paraíba	*Validação da nomenclatura de diagnósticos, resultados e intervenções de enfermagem para a clínica cirúrgica do Hospital Universitário da UFPB*	Master’s Dissertation
E6^(19)^	2018/BVS	Brazil/University of Campinas	*Acurácia dos indicadores clínicos dos diagnósticos de enfermagem do subconjunto terminológico “ommunity nursing” para usuários hipertensos e diabéticos*	Doctoral Thesis
E7^(20)^	2019/BVS	Brazil/Federal University of Paraíba	*Validação da nomenclatura de diagnósticos, resultados e intervenções de enfermagem para a unidade de terapia intensiva geral do Hospital Universitário da UFPB*	Master’s Dissertation
E8^(21)^	2019/BVS	Brazil/Federal University of Paraíba	*Validação da nomenclatura de diagnósticos, resultados e intervenções de enfermagem para a clínica médica do Hospital Universitário Lauro Wanderley/UFPB*	Master’s Dissertation
E9^(22)^	2019/BVS	Brazil/Federal University of Paraíba	*Subconjunto terminológico da CIPE^®^ para pessoas com síndrome metabólica: base conceitual para a teoria de médio alcance do cuidado no contexto de risco cardiovascular*	Doctoral Thesis
E10^(23)^	2020/PubMed	Korea/Inha University	Development of ICNP-based inpatient falls prevention catalogue	Article
E11^(24)^	2021/BVS	Brazil/Federal Fluminense University	*Subconjunto terminológico da CIPE^®^ para pessoas com doença renal crônica em tratamento conservador*	Doctoral Thesis
E12^(25)^	2021/BVS	Brazil/Pontifical Catholic University of Paraná	*Validação clínica do subconjunto terminológico da CIPE^®^ para o autocuidado da pessoa com estomia de eliminação intestinal*	Master’s Dissertation
E13^(26)^	2021/BVS	Brazil/Pontifical Catholic University of Paraná	*Validação clínica do subconjunto terminológico CIPE^®^ cuidados paliativos para um morrer com dignidade*	Master’s Dissertation
E14^(27)^	2021/BVS	Brazil/Pontifical Catholic University of Paraná	*Aplicabilidade das intervenções de enfermagem do subconjunto terminológico da CIPE^®^ para assistência à mulher e à criança em processo de amamentação*	Master’s Dissertation
E15^(28)^	2022/BVS	Brazil/Federal University of Paraíba	*Aplicabilidade clínica do subconjunto terminológico da CIPE^®^ para a mulher idosa com vulnerabilidade relacionada ao HIV/AIDS: desenvolvimento de uma teoria de médio alcance*	Doctoral Thesis

In terms of production type, the studies consisted of seven master’s dissertations (46.7%), seven doctoral theses (46.7%), and one scientific article (6.7%). Regarding research focus, 86.7% centered on adult and elderly health, while only two studies addressed maternal and child health (13.3%). Concerning the origin of the studies, eight were conducted at the Federal University of Paraíba (UFPB), linked to the Brazilian ICNP^®^ Center (53.3%).

An analysis was conducted to determine the type of theoretical framework or concept that underpinned the methodology, sample, follow-up, data collection technique, and analysis, with these data represented in Chart 3. It was found that ten studies employed case studies (66.7%), two did not present any references (13.3%), one was an accuracy study (6.7%), one was a case-control study (6.7%), and one involved clinical face validation (6.7%). Regarding the sample size, it ranged from five to 25 cases. Follow-up varied, including visits/consultations and other forms of monitoring. The data collection technique was based on the phases of the NP, and the analysis was organized using conceptual maps, single-case analyses, or simple statistics.

**Chart 3:** Characterization of studies according to framework/concept, sample, segment, technique, and analysis, Niterói, Rio de Janeiro, Brazil, 2024

ID	Framework/Concept	Sample	Follow-up	Data Collection Technique	Analysis	Level of Evidence/Degree of Recommendation	Theoretical Support
E1	Case Study	5 cases	12 meetings	Nursing consultation/Focused interview/Direct observation	Conceptual maps/Single case	4/D	Life Model Theory
E2	Case Study	5 cases	5 meetings	Nursing Process	Conceptual map	4/D	Comfort Theory
E3	Case Study	6 cases	3 meetings	Nursing Process	Nursing Process	4/D	Basic Human Needs Theory
E4	Case-Control Study	3,244 consultations	Retrospective	Nursing Process	Statistics	4/D	Environmental and Occupational Nursing Theory
E5	Case Studies	25 cases	4 visits per case over 5 days	Nursing Process	Collective case	4/D	Basic Human Needs Theory
E6	Diagnostic Accuracy	363 participants	-	Nursing Process	Simple statistics	4/D	Not Identified
E7	Multiple Case Study	7 cases	7 days	Nursing Process	Described each case	4/B	Basic Human Needs Theory
E8	Case Study	20 cases	10 visits	Nursing Process	Single case	4/D	Basic Human Needs Theory
E9	Case Study	22 cases	Not identified	Nursing Process	Simple statistics	4/D	Basic Human Needs Theory
E10	Face Clinical Validation	12 specialists	Not identified	Questionnaire	9-point scale	5/D	Not Identified
E11	Not Applicable	12 cases	1 consultation	2 nurses	Reliability	4/D	Adaptation Theory
E12	Case Study	9 cases	2 to 11 visits	Nursing Process	Simple statistics	4/D	General Theory of Self-Care
E13	Case Study	20 cases	Prospective	Nursing Process	Simple statistics	4/D	Theory for the Preservation of Dignity
E14	Not Applicable	15 postpartum women/6 nurses and 17 nursing technicians	Cross-sectional/systematic and non-participatory observation	Instrument with interventions	Simple statistics	4/D	Interactive Breastfeeding Theory
E15	Case Study	9 cases	Applied Research	Nursing Process	Case	4/D	General Theory of Self-Care

**Figure 2:**
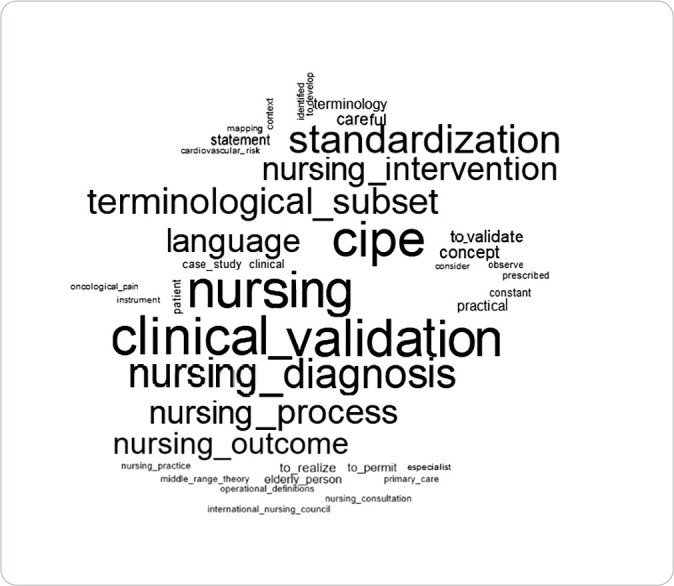
Word Cloud of the Textual Corpus from Theses and Dissertations that Conducted Clinical Validation of Terminological Subsets (n=15), Niterói, Rio de Janeiro, Brazil, 2024

The results obtained from the word cloud indicated that the most relevant terms were “nursing”, “clinical validation”, and “nursing diagnosis”, reflecting the essence of the ICNP that supports the system. Other terms appeared with lower frequency, such as “language”, “standardization”, “validate”, “case study”, “terminology”, “statement”, and “care”, highlighting the guiding principles that shape the current perspectives of nursing studies (Figure 2).

**Figura 3:**
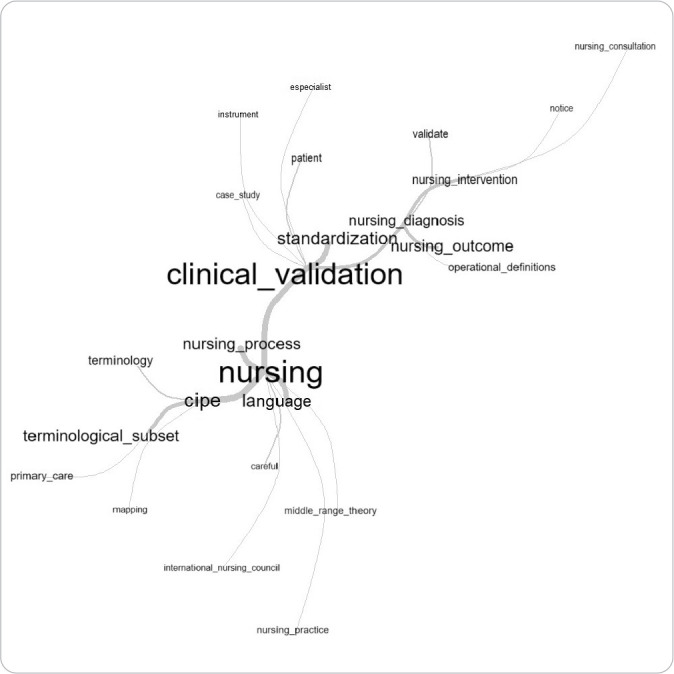
Árvore de similitude do corpus textual dos resumos de teses e dissertações que realizaram validação clínica de subconjuntos terminológicos (n=15), Niterói, Rio de Janeiro, Brasil, 2024

In the structure of the similarity tree, the central word “clinical validation” reflects a strong relationship with the terms “nursing process” and “nursing”, which are connected to a network of words such as “middle-range theory”, “instrument”, “validate”, “observe”, “nursing practice”, and “patient”, as illustrated in Figure 3.

## DISCUSSION

This review presents originality in its purpose, as it successfully identified comprehensive studies and integrated the principles of clinical validation, contributing to a synthesis of knowledge regarding the methodological relevance and the outcomes that ICNP^®^ can offer in clinical practice. The analysis highlighted, through the word cloud, the central role of Brazil as the primary producer of clinical validation studies, with a notable emphasis on the Federal University of Paraíba (UFPB). Review studies confirm the pioneering role and prominence of UFPB, as it houses the ICNP^®^ Research and Development Center, which has maintained a strong scientific direction over the past 17 years since its establishment^(29-31)^.

Another noteworthy institution is the Pontifical Catholic University of Paraná, which conducted three clinical validation studies. This institution offers a Graduate Program in Health Technology, including master’s and doctoral degrees with research lines that cover health informatics, among other topics, such as nursing terminologies and standards for record-keeping. These data reflect the growth of postgraduate programs, as they establish networks of researchers with common interests, continuing and strengthening research and scientific production in the country^(32)^. It is worth noting that this program has ongoing studies related to the clinical validation of subsets.

It is also worth highlighting the inclusion of the theme in other institutions, as seen in this study, which promote lines of research associating standardized language systems in nursing and technologies with the care process. This fact reflects the growth of academic production in the country through postgraduate programs and by reflecting on the development and implementation of technological production and innovation to qualify nursing services in various fields^(29)^. Therefore, conducting clinical validation studies is necessary, since it is an advance in the studies of ICNP^®^ evidence analysis in its practical dimension, since content analysis studies are more developed and guided by the method.

A beneficial aspect of the research relates to studies E6 and E13, which investigated phenomena related to diagnoses and interventions, respectively, without validating the complete subset. The terminology and definitions of ICNP^®^ are generally developed through deductive and inductive processes, often without the empirical support of scientific evidence seen in other language systems. ICNP^®^ is recognized for its greater productive autonomy, relying on terms predominantly associated with signs, symptoms, or clinical situations, thereby aligning its statements more closely with clinical practice and promoting significant advances in practice documentation through the implementation of standards that facilitate terminological standardization^(3)^. Thus, the importance of studies validating phenomena, including nursing outcomes, is emphasized, as they provide mechanisms indicating responses to clinical judgments and their impact on individuals.

As seen, the studies found used clinical case studies, in line with the ICN recommendations. The results from the case studies were presented individually and analyzed as a single case or collectively, emphasizing the most frequently identified statements of diagnoses, results and nursing interventions.

Although the ICN recommends the implementation of case studies, there is limited exploration of their developments. The use of the term “case study” raises questions about its application context. Researchers may view it in various contexts: as a research method, a technique, an instrument, or an approach, serving, for example, to problematize the relationship between theory and practice or in medical and psychological studies that reflect dynamics or specific pathologies^(33)^.

A seminal and precedent-setting study on case studies defines it as an analysis of a specific system or case, conducted through meticulous data collection from various sources of information. Similarly, it is classified as a detailed investigation of a unit, group, or individual, taking into account its inherent complexity and dynamism, and providing pertinent information for the decision-making process^(34)^. As noted, although some aspects may be unclear, it is understood that case studies can indeed be characterized as a deep and relevant approach in line with the proposal for terminological subsets.

However, the studies found used the concepts of the main and current author on case study, Robert Yin, which is in line with a review conducted^(33)^. For this author, the case study is an empirical research that explores in detail a current phenomenon, considering the case in its real context. This method is broad, encompassing everything from the elaboration of the research project and the definition of its elements to the techniques used for data collection and specific approaches for analyzing the information^(35)^. The complexity of this method is determined by theoretical supports that serve as guidance for the researcher’s work. Therefore, this is a modality used in the biomedical and social sciences, resulting from an in-depth and exhaustive study, in order to expand and detail knowledge^(36)^.

Case studies can integrate qualitative and quantitative methods, and are used in various circumstances to investigate individual, collective, organizational, political and social phenomena. This approach allows researchers to analyze a case comprehensively, considering its real context. Studies are divided into two types: single case study and multiple case study. The second type covers more than one case and offers the advantage of allowing a more solid analysis through the evidence collected^(37)^.

In research protocols, it is crucial to ensure reliability by providing sufficient information so that, when repeated under the same conditions, the study can yield the same results. The study protocol should include the following topics: an overview of the research - objective, research question, guiding literature, theoretical model; procedures adopted for data collection - document analysis, interviews, field observation, data collection instruments; procedures to address unforeseen circumstances during data collection; necessary resources; a timeline for data collection activities; a plan for analyzing the collected data with a breakdown of the nature of the information; and a guide for the report^(37)^.

Theoretically, none of the studies adopted common criteria, whether theoretical or conceptual, for the operationalization of case studies, except for the adoption of Yin’s framework. Therefore, it is necessary to return to the literature on the subject in order to recognize criteria that can be used in the structuring of a terminological subset. Given the aspects presented so far, it is necessary to associate how they can be adapted to the clinical validation of a terminological subset, in order to contribute to the impact repercussions of the final product. It is still unclear which is the most coherent way of presenting and analyzing these cases.

This allows for diverse methodological structures within case studies. Study 4 adopted a methodological approach with a case-control study design to clinically validate the subset in the area of occupational health. In this study, an association was observed between variables used in occupational history-taking and nursing diagnoses/results, defining “cases” as workers presenting nursing diagnoses/results in three theoretical classes simultaneously and “controls” as those covered by fewer than three theoretical classes.

Another study, Study 6, employed diagnostic accuracy to evaluate the relationship between a diagnostic test and a reference standard. This study was innovative in using such a method in conjunction with ICNP^®^, identifying diagnostic statements and their respective clinical indicators^(38)^. Study 11 applied the concepts of relevance and applicability through dual evaluation by nurses regarding the presence of a diagnostic statement, analyzed using the Kappa (k) concordance coefficient^(39)^.

Research that reviewed ICNP^®^ validations shows similarities with current research in showing that there are strategies that are used for clinical validation and that build robust research possibilities^(40)^. As an example, there are two Italian studies that carried out multicenter and cross-sectional studies for nursing diagnoses similar to the Brazilian method^(41,42)^. However, these studies did not use the term clinical validation, which is why they were not included in the current review.

Currently, research in nursing has focused on finding ways to integrate evidence into practice, particularly through tools that support the work of nurses and their teams. The goal is to simplify decision-making by providing detailed descriptions of specific care situations, with precise information on how to act. This results in greater safety for the team, reduces variation in actions taken, facilitates the adoption of new technologies, and promotes efficient resource use. Furthermore, these approaches enable the monitoring of process and outcome indicators, contributing to the continuity of services and the evaluation of the quality and safety of care provided^(43)^.

Study 10 presented a method of “face clinical validation” with professionals specializing in fall prevention. This raises questions about the meaning of the term “clinical”, as it may refer to the clinical experience of professionals validating based on their practice or be confused with the expected clinical validation for this study. Despite this dichotomy, the study was included due to its use of the term, providing greater clarification and expansion of the method. In this context, it is understood that alternatives involving new models of clinical validation contribute to overcoming limitations, enhancing nursing language systems, reducing discrepancies, and increasing nurses’ ability to appropriately recognize nursing phenomena presented by individuals^(44)^.

It is noted that in the three initial productions, the number of patients in the sample was small, which was not theoretically justified by the authors. It is assumed that the most commonly used type of sample, the non-probabilistic convenience sample, was adopted without any criteria being set out. Although these and some other studies found did not adopt any statistical method, the sampling issue is fundamental and needs to be seen to the point of demonstrating its significance and credibility of the subset.

It is expected that with a larger sample size, the greater will be the diversification of the nurse’s clinical judgment and consequently a greater impact on care practices. Therefore, what is expected is that quick studies are more common, which can support clinical practice, but which require some scientific result that promotes validity in decision-making^(45)^. Regardless of the sample size, the criteria adopted need to be clearer, methodological and personal limitations should be pointed out, since the academic chronology allows for comfortable recruitment and data analysis.

The studies met the recommendations regarding clinical validation being performed using the phases of the nursing process, that is, most of them adopted instruments capable of meeting the phases of this process, such as assessment and physical examination instruments or forms, and the validated subset itself. As described by the authors, the validation of the terminological subset must present methodological rigor, following a structured method, based on the five stages of the NP, and a conceptual standard that makes it possible to support this elaboration^(2)^.

The NP is understood as a methodological tool that requires cognitive, technical, and interpersonal interaction skills. It should be developed and implemented according to the needs of the individual, family, or community requiring professional attention, with an emphasis on intentional problem-solving^(46)^. Clearly, the primary identity of nursing resides in care, implemented through the NP, which guides actions and thought processes, enabling the documentation of professional practice^(47)^.

Therefore, clinical validation with the NP stages becomes fully enriching, strengthening a science and valuing its professionals. NP is the method that guides critical thinking and clinical judgment, necessary to structure the administration of nursing care, record care and highlight the activities performed by this team in services that often go unnoticed^(48)^.

The similarity tree highlights the importance of clinical validation derived from academic productions and reflects the impact of positioning nursing science within a standard applicable to real-world settings. Thus, the clinical validation phase becomes essential to strengthen the application of ICNP^®^ by clinical nurses, fostering the integration between academic knowledge and clinical practice^(49)^. However, within this context, the term “clinical applicability” emerges in Studies 9, 14, and 15 as a central axis for labeling clinical validation studies, even replacing it in some study conceptualizations^(49)^. This adoption may have been designed to emphasize the importance of transferring care technology to practice. Evidence-based science is becoming increasingly relevant in this dynamic and challenging scenario, where research outcomes are essential for questioning, supporting, and transforming approaches to education, care, and process management^(50)^.

It is also noted that the decision to prove that the statement is applicable, when it is applied at least once, at a given moment in the act of caring, is present, which justifies its clinical applicability^(49)^. Another form of validation was the agreement between specialist nurses tested by obtaining coefficients on interobserver reliability^(39)^. Regardless of the way the study is named, the need for further clarification is relevant, since such studies have always been classified as validation, even though statistical indexes were not used.

### Study limitations

This study has some limitations. First, the initially large number of productions was significantly reduced, highlighting Brazil as the sole producer of clinical applicability studies related to subsets, which cannot be generalized, as there is a need for deeper exploration of language systems and methods used across different continents. Second, some references were not found during the review, requiring the authors to seek information and the complete text of the productions directly from the online pages of postgraduate programs. This may be related to the fact that international authors use different terms instead of “clinical validation”, employing other research methods. Finally, it is believed that due to the variety of methodological steps or the insufficient explanation of these in the studies, some information was not found clearly, which may lead to weaknesses in the results found. Furthermore, academic productions must be disseminated through scientific articles in order to publicize aggregated and easily accessible results.

### Contributions to the Field of Nursing

The main motivation for understanding validation studies is to apply them in clinical practice, thereby creating a positive impact on the health demands of the population. In other words, it is necessary to “translate” this knowledge, which is satisfactorily achieved in the theoretical domain but remains underutilized in everyday nursing practice. Therefore, this is a dynamic movement that promotes continuous advancements and requires understanding to generate new and promising perspectives. With this, language systems in nursing are strengthened and reveal their importance as the basis of the nursing process and the valorization of scientific knowledge in the area.

## CONCLUSIONS

This study identified characteristics and studies related to the clinical validation of ICNP® terminological subsets. Academic production has shown growth, primarily focusing on adult and elderly health. The results indicate that Brazil is the leading producer in this field, with a notable emphasis on the Northeast region, which has adopted case studies as the main methodological framework. Case analyses or statistical analyses were the methods most commonly used, but they lack progress, since the studies are incipient and require availability and greater uniformity of methods. This issue can make it difficult to discuss the reliability of the findings, given that the same indicators end up being validated for different groups, for example, but through completely different methodological phases.
