# Supplementary material for: Clinical validation of terminological subsets of the international classification for nursing practice: a scoping review
Source: Rev Bras Enferm. 2025 Apr 28;78(2):e20240203. doi: 10.1590/0034-7167-2024-0203 (PMC12037189; doi:10.1590/0034-7167-2024-0203)
Supplement: Translation

# Translation: Validação clínica de subconjuntos terminológicos da Classificação Internacional para a Prática de Enfermagem: revisão de escopo

**DOI:** 10.1590/0034-7167-2024-0203pt

**Authors:** Fernanda de Freitas Ferreira, Silvia Maria de Sá Basílio Lins, Harlon França de Menezes, Rebecca Stefany da Costa Santos, Juliana Otaciana dos Santos, Richardson Augusto Rosendo da Silva

**Affiliations:** I Universidade Federal Fluminense. Niterói, Rio de Janeiro, Brasil; II Universidade Federal do Rio Grande do Norte. Natal, Rio Grande do Norte, Brasil

**Keywords:** Processo de Enfermagem, Estudo de Validação, Terminologia Padronizada em Enfermagem, Diagnóstico de Enfermagem, Pesquisa em Enfermagem Clínica

## Abstract

**Objetivos:**

mapear as evidências científicas sobre a validação clínica de subconjuntos terminológicos da Classificação Internacional para a Prática de Enfermagem (CIPE^®^).

**Métodos:**

revisão de escopo realizada entre janeiro e fevereiro de 2024 em nove bases de dados. A elaboração seguiu as diretrizes estabelecidas pelo JBI, bem como o checklist *Preferred Reporting Items for Systematic Reviews and Meta-Analyses Extension for Scoping Reviews.* A análise dos dados foi realizada por meio de estatística descritiva simples e análise lexical, utilizando o software IRaMuTeQ.

**Resultados:**

foram selecionados 15 estudos, sendo a maioria produzida no Brasil (93,3%); sete estudos de dissertação (46,7%); 86,7% tinham como foco a saúde do adulto e idoso, e 13,3% referiam-se à saúde materno-infantil. Oito estudos foram realizados na Universidade Federal da Paraíba (53,3%) e dez produções adotaram estudos de caso (66,7%).

**Conclusões:**

observa-se uma carência de avanços, uma vez que os estudos são incipientes e requerem maior disponibilidade e uniformidade de métodos.

## INTRODUÇÃO

Para a construção do Processo de Enfermagem (PE), o uso de sistemas de linguagens padronizadas na prática assistencial contribui para o raciocínio clínico e para a tomada de decisões com o objetivo de melhorar a saúde e as respostas humanas dos pacientes. Nesse contexto, identifica-se a relevância dos indicadores clínicos para os diagnósticos de enfermagem, que constituem evidências fundamentais para a identificação e a aplicação de intervenções. Diagnósticos de enfermagem precisos e validados orientam a escolha de intervenções adequadas, capazes de produzir os resultados esperados, subsidiando pesquisas, gestão, educação e a elaboração de propostas de políticas públicas^(1,2)^.

Pesquisas indicam que a adoção de uma linguagem padronizada na Enfermagem visa à consolidação dessa profissão como uma ciência, estabelecendo um cuidado sistematizado que se fundamenta em evidências e se orienta pelas prioridades de saúde pertinentes ao seu campo de atuação. Apenas por meio de uma terminologia específica e uniforme, os profissionais de enfermagem poderão demonstrar seu papel científico no âmbito dos cuidados à saúde, utilizando essa linguagem nas diversas fases do PE^(3,4)^.

Com a publicação da recente Resolução do Conselho Federal de Enfermagem 736 de 2024, fica claro que os sistemas de linguagens padronizadas apoiam o PE, baseando-se nos melhores níveis de evidências científicas. Os sistemas de linguagem possuem conceitos a serem criados, estudados e validados, motivo pelo qual o processo de validação é indispensável para aperfeiçoá-los e autenticá-los, com vistas a desenvolver um conhecimento dinâmico e investigações que envolvam novas ferramentas na possibilidade de aplicá-lo no cotidiano do profissional que o produz^(5,6)^.

Nesse sentido, diferentes tipos e métodos de validação são aplicados, como as validações de conteúdo, conceito, semântica, construto, critério e clínica. Entre essas, a validação clínica tem sido aprofundada e utilizada como parte do processo metodológico em pesquisas. Portanto, torna-se urgente a discussão sobre os principais aspectos para o seu desenvolvimento, uma vez que é uma etapa relevante para a construção desses estudos e aponta para a necessidade de aprimoramento^(7)^.

As orientações metodológicas do Conselho Internacional de Enfermeiras (CIE) destacam que os enunciados de diagnósticos, resultados e intervenções de enfermagem, organizados no formato de Subconjuntos Terminológicos da Classificação Internacional para a Prática de Enfermagem (CIPE^®^), devem ser validados clinicamente. Essa validação deve ser realizada com base nas fases do PE: avaliação, diagnóstico, planejamento, implementação e evolução^(1)^.

Contudo, o método brasileiro não inclui a etapa de validação clínica do subconjunto, embora a considere importante e incentive estudos com o objetivo de validar clinicamente o subconjunto construído. Dessa forma, os estudos desse tipo geralmente estão vinculados a conceitos diagnósticos, de resultados ou de intervenções de enfermagem, e não a um subconjunto terminológico. Tal fato estabelece uma lacuna no conhecimento que precisa ser abordada por pesquisadores da área^(1)^.

Portanto, com o objetivo de compreender o que já foi escrito sobre a validação clínica e seu impacto nos subconjuntos terminológicos da CIPE^®^, este estudo foi idealizado como um potencial instrumento norteador para pesquisadores que desejam aprimorar e validar os dados de sua prática, oferecendo subsídios para a divulgação e produção de conhecimento.

## OBJETIVOS

Mapear as evidências científicas sobre a validação clínica de subconjuntos terminológicos da CIPE^®^.

## MÉTODOS

### Aspectos éticos

Por se tratar de uma pesquisa de revisão, não foi necessária avaliação ética, uma vez que o material utilizado é de domínio público e não envolve a participação de seres humanos.

### Desenho do estudo

A presente análise configura uma revisão de escopo, organizada em conformidade com as diretrizes do guia internacional *Preferred Reporting Items for Systematic Reviews and Meta-Analyses* (*PRISMA*)^(8)^ e o método sugerido pelo *JBI* ^(9)^. Revisões desse tipo têm como objetivo principal traçar um panorama dos conceitos que fundamentam uma determinada área do conhecimento, avaliar a profundidade, extensão e natureza da pesquisa realizada, compilar e disseminar os dados obtidos e identificar as lacunas existentes nas pesquisas^(9)^. O protocolo foi registrado no *Open Science Framework* (*OSF*), disponível em (https://doi.org/10.17605/OSF.IO/DWYKV ).

### Estratégia de busca e fonte de dados

Para cumprir as etapas deste estudo, foram definidos o objetivo do estudo e a questão de pesquisa, de acordo com a combinação mnemônica PCC: P (*Population*) - Artigos publicados sobre subconjuntos terminológicos; C (*Concept*) - Métodos de validação clínica; C (*Context*) - Estudos realizados em qualquer país e tipo de instituição. Com base nessas definições, estabeleceu-se o objetivo do estudo e a seguinte questão norteadora: quais são os métodos utilizados para a validação clínica de subconjuntos terminológicos da CIPE^®^ na literatura mundial?

Na elaboração da estratégia de busca, foram utilizados descritores controlados do *Medical Subject Headings* (*MeSH*) e dos Descritores em Ciências da Saúde (DeCS), assim como palavras-chave, com o objetivo de ampliar o material disponível na literatura. Para isso, empregaram-se também os operadores booleanos “*OR*” e “*AND*”, além da seleção de sinônimos contidos no *MeSH* e DeCS. A formulação da estratégia de busca foi realizada em colaboração com um bibliotecário do Conselho Regional de Enfermagem de Minas Gerais (COREN-MG). Os DeCS/*MeSH* utilizados foram: Terminologia Padronizada em Enfermagem e CIPE^®^, com a aplicação do operador booleano “*OR*”: “Terminologia Padronizada em Enfermagem” *OR* “*Standardized Nursing Terminology*” *OR* “*Terminología Normalizada de Enfermería*” *OR* “CIPE^®^” *OR* “ICNP^®^” (Quandro 1).

A investigação foi realizada entre janeiro e fevereiro de 2024, abrangendo as seguintes bases de dados, repositórios e diretórios: MEDLINE (via *PubMed*) e CINAHL (por meio da plataforma *EBSCO*). Em seguida, foi conduzida uma busca mais ampla, utilizando as mesmas palavras-chave e critérios de busca nas bases de dados *Web of Science, Science Direct* (via plataforma *Scopus*), *Cochrane Database of Systematic Reviews*, além do portal Biblioteca Virtual em Saúde (BVS), da Biblioteca Digital Brasileira de Teses e Dissertações (BDTD) e do Instituto Brasileiro de Informação em Ciência e Tecnologia (IBICT), e na plataforma *Science.gov*, para a exploração da literatura cinzenta.

### Coleta, organização e análise dos dados

Os estudos completos foram considerados para a pesquisa a partir da leitura dos títulos por dois autores independentes. Após a leitura dos resumos, foi realizada uma pré-seleção dos estudos que se enquadravam nos critérios de inclusão desta revisão. Em casos de dúvidas ou discordâncias quanto à análise dos títulos, resumos e relevância dos estudos, a orientadora do estudo foi consultada. Utilizou-se o software Rayyan^®^, que permitiu a verificação de artigos duplicados, a inserção de rótulos explicativos e justificativas para exclusão e inclusão.

O refinamento dos estudos foi baseado nos critérios de elegibilidade estabelecidos. Foram incluídos estudos sobre CIPE e validação, disponíveis gratuitamente na íntegra. Excluíram-se os estudos que não abordassem métodos de validação clínica. Optou-se por não adotar recorte temporal nem idiomático, a fim de abranger a totalidade dos estudos.

Os dados foram extraídos, organizados e caracterizados em planilhas do *Microsoft Office Excel* ^®^, seguindo a seguinte ordem: base de dados, ano de publicação, local de publicação, referencial/conceito, amostra, seguimento, técnica de coleta, análise, nível de evidência e grau de recomendação, além de suporte teórico. Para o nível de evidência (NE) e grau de recomendação, foram utilizadas as prerrogativas do *Oxford Centre for Evidence-Based Medicine* ^(10)^.

Em seguida, os dados foram analisados de duas maneiras: 1) por estatística descritiva (relativa e absoluta), apresentada em quadros; e 2) pela análise lexical, realizada com o uso do software *Interface de R pour les Analyse Multidimensionnelles de Textes et de Questionnaires* (IRaMuTeQ), versão 0.7 Alpha 2, e *R* versão 3.2.3, que orienta uma perspectiva quantitativa para dados de cunho qualitativo^(11)^.

Para compor o corpus textual, foram incluídos os resumos dos estudos, a partir dos quais foram apresentados os Segmentos de Texto (ST) e o agrupamento das palavras mais significativas ^(11)^. Entre os métodos de análise permitidos pelo software, optou-se pela apresentação dos resultados deste estudo por meio das seguintes análises: 1) Nuvem de palavras, que representa graficamente as ocorrências do corpus textual, indicando que cada palavra tem um tamanho proporcional à sua frequência, expressa por meio de uma figura que não apresenta dados descritivos, mas permite uma rápida identificação dos termos com maior significado no conjunto dos textos^(12)^; 2) Árvore de Similitude, elaborada com base nas ocorrências das palavras presentes nos segmentos de texto^(13)^. Essa análise demonstra de forma visual a origem e a conexão das palavras. Ambas as análises foram discutidas com apoio da literatura.

## RESULTADOS

Foram identificados 2.953 estudos nas bases de dados, dos quais 1.551 foram removidos por estarem duplicados. Restaram 1.402 produções, e, após a leitura dos títulos e resumos, 1.273 foram excluídas por não atenderem aos critérios de inclusão. Selecionaram-se 129 artigos para leitura integral, resultando na exclusão de 85 textos que não abordavam subconjuntos terminológicos da CIPE. Uma nova rodada de leitura integral foi realizada com 44 artigos, culminando na exclusão de 29 produções que não abordavam a validação clínica. Restaram, portanto, 15 produções, que constituem o objeto desta revisão de escopo.

A Figura 1 especifica os resultados das etapas da análise, seguindo o modelo *PRISMA Flow Diagram*. A análise das publicações quanto ao ano de publicação, base de dados, país, instituição, título e tipo de produção foi sintetizada no Quadro 2. Optou-se por numerar e ordenar os estudos por data crescente de publicação, com a letra (E) representando “estudo”, seguida do número correspondente. Em relação ao ano de publicação, observou-se que o primeiro estudo foi publicado em 2014, com um aumento progressivo a partir de 2016. O Brasil foi o país que mais produziu estudos de validação clínica (93,3%).

**Quadro 1:** Estratégia de busca, Niterói, Rio de Janeiro, Brasil, 2024

Fonte de dados	Estratégia de busca adotada
BVS	(“Terminologia Padronizada em Enfermagem” *OR* “*Standardized Nursing Terminology*”) *OR* “*Terminología Normalizada de Enfermería*” *OR* CIPE OR ICNP; “Estudos de Validação como Assunto” *OR* “*Validation Studies as Topic*” *OR* “*Estudios de Validación como Asunto*” *OR* “Estudo de Validação” *OR* “*Validation Study*” *OR* “*Estudio de Validación*”
PUBMED	“*Standardized Nursing Terminology*” *OR* “*ICNP Terminology*” *OR* “*Validation Studies as Topic*” *OR* “*Validation Study*” *OR Validation*

*BVS - Biblioteca Virtual em Saúde.*

**Quadro 2:** Caracterização dos estudos segundo código E1 a E14, ano, base de dados, país e instituição de publicação, título e tipo de produção relacionados com o tema do estudo, Niterói, Rio de Janeiro, Brasil, 2024

ID	Ano/ Base	País/Instituição	Título	Tipo de produção
E1^(14)^	2014/BVS	Brasil/ Universidade Federal da Paraíba	Validação do subconjunto terminológico da CIPE^®^ para a pessoa idosa	Tese de Doutorado
E2^(15)^	2016/BVS	Brasil/ Universidade Federal da Paraíba	Validação do Subconjunto Terminológico CIPE^®^ para pacientes com dor oncológica	Tese de Doutorado
E3^(16)^	2016/BVS	Brasil/ Universidade Federal da Paraíba	Validação da nomenclatura de diagnósticos, resultados e intervenções de enfermagem para a clínica pediátrica do Hospital Universitário da UFPB	Dissertação de Mestrado
E4^(17)^	2017/BVS	Brasil/Universidade de Brasília	Subconjunto terminológico CIPE^®^ para a prática de enfermagem ambiental e ocupacional	Tese de Doutorado
E5^(18)^	2017/BVS	Brasil/ Universidade Federal da Paraíba	Validação da nomenclatura de diagnósticos, resultados e intervenções de enfermagem para a clínica cirúrgica do Hospital Universitário da UFPB	Dissertação de Mestrado
E6^(19)^	2018/BVS	Brasil/Universidade de Campinas	Acurácia dos indicadores clínicos dos diagnósticos de enfermagem do subconjunto terminológico *“community nursing”* para usuários hipertensos e diabéticos	Tese de Doutorado
E7^(20)^	2019/BVS	Brasil/ Universidade Federal da Paraíba	Validação da nomenclatura de diagnósticos, resultados e intervenções de enfermagem para a unidade de terapia intensiva geral do Hospital Universitário da UFPB	Dissertação de Mestrado
E8^(21)^	2019/BVS	Brasil/ Universidade Federal da Paraíba	Validação da nomenclatura de diagnósticos, resultados e intervenções de enfermagem para a clínica médica do Hospital Universitário Lauro Wanderley/UFPB	Dissertação de Mestrado
E9^(22)^	2019/BVS	Brasil/ Universidade Federal da Paraíba	Subconjunto terminológico da CIPE^®^ para pessoas com síndrome metabólica: base conceitual para a teoria de médio alcance do cuidado no contexto de risco cardiovascular	Tese de Doutorado
E10^(23)^	2020/PubMed	Coreia/*Inha University*	*Development of ICNP-based inpatient falls prevention catalogue*	Artigo
E11^(24)^	2021/BVS	Brasil/Universidade Federal Fluminense	Subconjunto terminológico da CIPE^®^ para pessoas com doença renal crônica em tratamento conservador	Tese de Doutorado
E12^(25)^	2021/BVS	Brasil/Pontifícia Universidade Católica do Paraná	Validação clínica do subconjunto terminológico da CIPE^®^ para o autocuidado da pessoa com estomia de eliminação intestinal	Dissertação de Mestrado
E13^(26)^	2021/BVS	Brasil/ Pontifícia Universidade Católica do Paraná	Validação clínica do subconjunto terminológico CIPE^®^ cuidados paliativos para um morrer com dignidade	Dissertação de Mestrado
E14^(27)^	2021/BVS	Brasil/ Pontifícia Universidade Católica do Paraná	Aplicabilidade das intervenções de enfermagem do subconjunto terminológico da CIPE^®^ para assistência à mulher e à criança em processo de amamentação	Dissertação de Mestrado
E15^(28)^	2022/BVS	Brasil/ Universidade Federal da Paraíba	Aplicabilidade clínica do subconjunto terminológico da CIPE^®^ para a mulher idosa com vulnerabilidade relacionada ao HIV/AIDS: desenvolvimento de uma teoria de médio alcance	Tese de Doutorado

**Figura 1:** Diagrama PRISMA referente ao processo de seleção dos estudos, 2023

Em relação ao tipo de produção, os estudos se dividiram em sete dissertações de mestrado (46,7%), sete teses de doutorado (46,7%) e um artigo científico (6,7%). Quanto à área de pesquisa, 86,7% tinham como foco a saúde do adulto e do idoso, e apenas dois estudos referiam-se à saúde materno-infantil (13,3%). Sobre a origem das produções, oito estudos foram realizados na Universidade Federal da Paraíba (UFPB), vinculados ao Centro CIPE^®^ brasileiro (53,3%).

Quis-se saber qual seria o tipo de referencial ou conceito que embasou a metodologia, amostra, seguimento, técnica de coleta de dados e análise, estando estes dados representados no Quadro 3. Verificou-se que dez estudos adotaram estudos de caso (66,7%), dois não apresentaram referências (13,3%), um estudo de acurácia (6,7%), um caso-controle (6,7%) e outro validação clínica de face (6,7%). Sobre a amostra, ficou entre cinco e 25 casos. O seguimento foi variado com visitas/consultas a outras formas de acompanhamento. A técnica de coleta de dados se baseou nas etapas do PE e a análise foi organizada por mapas conceituais, caso único ou estatísticas simples.

**Quadro 3:** Caracterização dos estudos conforme referencial/conceito, amostra, segmento, técnica e análise, Niterói, Rio de Janeiro, Brasil, 2024

ID	Referencial/Conceito	Amostra	Seguimento	Técnica de coleta	Análise	Nível de Evidência/Grau de Recomendação	Suporte Teórico
E1^(14)^	Estudo de caso	5 casos	12 encontros	Consulta de enfermagem/ Entrevista focal/Observação direta	Mapas conceitual/ Caso único	4/D	Teoria do Modelo de Vida
E2^(15)^	Estudo de caso	5 casos	05 encontros	Processo de Enfermagem	Mapa conceitual	4/D	Teoria do Conforto
E3^(16)^	Estudo de Caso	6 casos	03 encontros	Processo de Enfermagem	PE	4/D	Teoria das Necessidades Humanas Básicas
E4^(17)^	Estudo de caso-controle	3.244 consultas	Retrospectivo	Processo de Enfermagem	Estatísticas	4/D	Teoria de Enfermagem Ambiental e Ocupacional
E5^(18)^	Estudo de casos	25 casos	04 visitas por caso em 05 dias	Processo de Enfermagem	Caso coletivo	4/D	Teoria das Necessidades Humanas Básicas
E6^(19)^	Acurácia diagnóstica	363 participantes	-	Processo de Enfermagem	Estatísticas simples	4/D	Não identificado
E7^(20)^	Estudo de caso múltiplos	7 casos	07 dias	Processo de Enfermagem	Descreveu cada caso	4/B	Teoria das Necessidades Humanas Básicas
E8^(21)^	Estudo de Caso	20 casos	10 visitas	Processo de Enfermagem	Caso único	4/D	Teoria das Necessidades Humanas Básicas
E9^(22)^	Estudo de Caso	22 casos	Não identificado	Processo de Enfermagem	Estatísticas simples	4/D	Teoria das Necessidades Humanas Básicas
E10^(23)^	Validação clínica de face	12 especialistas	Não identificado	Questionário	Escala de 9 pontos	5/D	Não identificado
E11^(24)^	Não aplicado	12 casos	01 consulta	02 enfermeiros	Confiabilidade	4/D	Teoria da Adaptação
E12^(25)^	Estudo de Caso	9 casos	02 a 11 visitas	Processo de Enfermagem	Estatísticas simples	4/D	Teoria Geral do Autocuidado
E13^(26)^	Estudo de Caso	20 casos	Prospectivo	Processo de Enfermagem	Estatísticas simples	4/D	Teoria para a preservação da dignidade
E14^(27)^	Não aplicado	15 puérperas/6 enfermeiros e 17 técnicos de enfermagem	Transversal/ observação, sistemática e não participativa	Instrumento com intervenções	Estatísticas simples	4/D	Teoria Interativa de Amamentação
E15^(28)^	Estudo de Caso	09 casos	Pesquisa Aplicada	Processo de Enfermagem	Caso	4/D	Teoria Geral do Autocuidado

Os resultados obtidos a partir da nuvem de palavras indicaram que os termos mais relevantes foram “enfermagem”, “validação clínica” e “diagnóstico de enfermagem”, trata-se da essência da CIPE que amparam o sistema. Outras palavras apresentaram menor frequência, como “linguagem”, “padronização”, “validar”, “estudo de caso”, “terminologia”, “enunciado” e “cuidado”, destacando as diligências norteadoras que regem as atuais perspectivas dos estudos de enfermagem (Figura 2).

**Figura 2:** Nuvem de palavras do corpus textual dos resumos de teses e dissertações que realizaram validação clínica de subconjuntos terminológicos (n=15), Niterói, Rio de Janeiro, Brasil, 2024

Na estrutura da árvore de similitude, a palavra central “validação clínica” reflete uma relação robusto com a palavra “processo de enfermagem” e “enfermagem”, que se conecta a uma rede de palavras, tais como “teoria de médio alcance”, “instrumento”, “validar”, “observar”, “prática de enfermagem” e “paciente”, o que pode ser observado na Figura 3.

**Figura 3:** Árvore de similitude do corpus textual dos resumos de teses e dissertações que realizaram validação clínica de subconjuntos terminológicos (n=15), Niterói, Rio de Janeiro, Brasil, 2024

## DISCUSSÃO

A presente revisão apresenta originalidade em seu propósito, pois foi capaz de buscar estudos abrangentes e agregar os preceitos da validação clínica, contribuindo para uma síntese de conhecimento sobre a relevância metodológica e os desdobramentos que a CIPE^®^ pode proporcionar nas práticas assistenciais. A análise evidenciou, por meio da nuvem de palavras, a centralidade do Brasil como o país que, de maneira quase exclusiva, produziu o maior número de estudos de validação clínica, com destaque para a UFPB. Estudos de revisão confirmam o pioneirismo e a notoriedade da UFPB, uma vez que lá se encontra o Centro de Pesquisa e Desenvolvimento da CIPE^®^, que tem forte direcionamento científico nos últimos 17 anos desde sua criação^(29-31)^.

Outra instituição que merece destaque é a Pontifícia Universidade Católica do Paraná, que apresentou três estudos de validação clínica. Essa instituição possui um Programa de Pós-Graduação em Tecnologia em Saúde, com oferta de cursos de mestrado e doutorado em linhas de pesquisa que incluem a temática de informática em saúde, abordando, entre outros assuntos, as terminologias e padrões de registro em enfermagem. Esse dado reflete a expansão da pós-graduação, pois articula redes de pesquisadores com interesses comuns, dando continuidade às pesquisas e fortalecendo o desenvolvimento da pesquisa e produção científica no país^(32)^. Ressalta-se que esse programa possui estudos em andamento sobre a validação clínica de subconjuntos.

Destaca-se também a inserção do tema em outras instituições, como visto neste estudo, que promovem linhas de pesquisas associando os sistemas de linguagem padronizada em enfermagem e tecnologias ao processo de cuidar. Esse fato reflete o crescimento da produção acadêmica no país por meio dos programas de pós-graduação e por refletirem sobre o desenvolvimento e implementação de uma produção tecnológica e de inovação para qualificar o serviço de enfermagem em diversos campos^(29)^. Com isso, a realização de estudos de validação clínica se faz necessário haja visto ser um avanço dos estudos de análises de evidências da CIPE^®^ em sua dimensão prática, já que os estudos de análise de conteúdo são mais desenvolvidos e guiados pelo método.

Um aspecto proveitoso nas pesquisas refere-se aos estudos E6 e E13, que investigaram fenômenos relacionados a diagnósticos e intervenções, respectivamente, sem validar o subconjunto completo. A terminologia e as definições da CIPE^®^ são geralmente desenvolvidas por processos dedutivos e indutivos, sem necessariamente contar com suporte comprobatório das evidências científicas, como ocorre em outros sistemas de linguagem. A CIPE^®^ é reconhecida por sua maior autonomia produtiva, baseando-se na utilização de termos predominantemente associados a sinais, sintomas ou situações clínicas, o que aproxima os enunciados da prática clínica e promove avanços significativos na documentação das práticas, por meio da implementação de normas que favorecem a padronização terminológica^(3)^. Assim, destaca-se a importância de estudos que validem os fenômenos, incluindo resultados de enfermagem, trazendo mecanismos que indiquem respostas aos julgamentos clínicos e seu impacto no indivíduo.

Como visto, os estudos encontrados se apropriaram dos estudos de caso clínicos, indo ao encontro com as recomendações do CIE. Os resultados provenientes dos estudos de casos foram apresentados de forma individual e analisados como um único caso ou coletivo, enfatizando-se os enunciados de diagnósticos, resultados e intervenções de enfermagem identificados com maior frequência.

Embora o CIE recomende a implementação de estudos de caso, há pouca exploração sobre seus desdobramentos. A utilização do termo “estudo de caso” gera dúvidas quanto ao contexto de aplicação. Pesquisadores podem considerar diversos contextos, como um método de pesquisa, uma técnica, um instrumento ou uma abordagem, servindo, por exemplo, para problematizar a relação entre teoria e prática, ou em estudos médicos e psicológicos que refletem dinâmicas ou patologias específicas^(33)^.

Um estudo precedente e seminal que aborda o estudo de caso conceitua-o como uma análise de um sistema específico ou de um caso, realizada por meio de uma coleta minuciosa de dados que abrange diversas fontes de informação. Da mesma forma, classifica-se como uma investigação detalhada de uma unidade, grupo ou indivíduo, considerando sua complexidade e dinamismo intrínsecos, e oferecendo informações pertinentes para o processo de tomada de decisão^(34)^. Como observado, embora esses aspectos possam ser pouco claros, compreende-se que o estudo de caso pode, de fato, caracterizar-se como uma abordagem profunda e condizente com a proposta dos subconjuntos terminológicos.

Porém, os estudos encontrados utilizaram os conceitos do autor principal e atual sobre estudo de caso, Robert Yin, que vem ao encontro de revisão realizada^(33)^. Para este, o estudo de caso é uma pesquisa empírica que explora detalhadamente um fenômeno atual, considerando o caso em seu contexto real. Esse método é amplo, englobando desde a elaboração do projeto de pesquisa e a definição de seus elementos até as técnicas utilizadas para a coleta de dados e as abordagens específicas para a análise das informações^(35)^. A complexidade deste método é determinada por suportes teóricos que servem de orientação ao trabalho do pesquisador. Por isso, esta é uma modalidade utilizada nas ciências biomédicas e sociais, resultantes de um estudo profundo e exaustivo, a fim de ampliar e detalhar o conhecimento^(36)^.

Os estudos de caso podem integrar métodos qualitativos e quantitativos, sendo utilizados em várias circunstâncias para investigar fenômenos individuais, coletivos, organizacionais, políticos e sociais. Essa abordagem possibilita que os pesquisadores analisem um caso de forma abrangente, considerando seu contexto real. Os estudos se dividem em dois tipos: estudo de caso único e estudo de casos múltiplos. O segundo tipo abrange mais de um caso e oferece a vantagem de permitir uma análise mais sólida através das evidências coletadas^(37)^.

No protocolo de pesquisa, é relevante para a obtenção da confiabilidade que as informações sejam fornecidas de modo que, quando repetido sob as mesmas condições, o estudo possa gerar os mesmos resultados. O protocolo do estudo deve conter os seguintes tópicos: visão geral da pesquisa - objetivo, questão de pesquisa, leituras norteadoras, modelo teórico; procedimentos adotados para coleta de dados - análise documental, entrevistas, observação em campo, instrumentos para coleta dos dados; procedimentos para lidar com imprevistos durante a coleta de dados; recursos necessários; agenda das atividades de coleta de dados; plano de análise dos dados coletados com discriminação da natureza das informações; e guia para o relatório^(37)^.

Teoricamente, nenhum dos estudos adotou critérios comuns, sejam eles teóricos/conceituais para a operacionalização dos estudos de casos, a não ser pela adoção do referencial de Yin. Deste modo, é preciso retornar à literatura sobre o tema a fim de reconhecer critérios que possam ser utilizados na estruturação de um subconjunto terminológico. Diante destes aspectos até aqui apresentados, é preciso associar como estes podem se adequar à validação clínica de um subconjunto terminológico, a fim de contribuir para repercussões de impacto do produto final. Ainda não se tem clareza de qual forma mais coerente de apresentação e análise destes casos.

Com isso, há margem para estruturas metodológicas distintas dentro do estudo de caso. O Estudo 4 adotou uma proposta metodológica com caráter de estudo caso-controle para validar clinicamente o subconjunto na área de saúde ocupacional. Verificou-se, nesse estudo, a associação de variáveis utilizadas na anamnese ocupacional com os diagnósticos/resultados de enfermagem, atribuindo-se como “casos” os trabalhadores que apresentaram diagnósticos/resultados de enfermagem em três classes teóricas simultaneamente, e como “controles” aqueles abrangidos por menos de três classes teóricas.

Outro estudo, o Estudo 6, utilizou a acurácia diagnóstica para avaliar a relação entre um teste diagnóstico e o padrão de referência. Esse estudo foi inovador ao empregar tal método em conjunto com a CIPE^®^, identificando os enunciados diagnósticos e seus respectivos indicadores clínicos^(38)^. O Estudo 11 utilizou os conceitos de pertinência e aplicabilidade por meio da avaliação em dupla de enfermeiros quanto à presença do enunciado diagnóstico, analisada pelo coeficiente de concordância de Kappa (k)^(39)^.

Pesquisa que revisou as validações da CIPE^®^ traz semelhanças com a pesquisa atual em mostrar que existem estratégias que são empregadas para validação clínica e que constroem possibilidades de pesquisa robustas^(40)^. Como exemplo, tem-se dois estudos italianos que realizaram estudos multicêntricos e transversais para diagnósticos de enfermagem tendo similitude ao método brasileiro^(41,42)^. Contudo, estes estudos não utilizaram do termo validação clínica, fato este que não foram incluídos na atual revisão.

Atualmente, as pesquisas na área de enfermagem têm se concentrado em encontrar maneiras de integrar evidências à prática, especialmente por meio de ferramentas que auxiliem o trabalho do enfermeiro e de sua equipe. O objetivo é tornar a tomada de decisões mais simples, fornecendo uma descrição detalhada de situações específicas de cuidado, com informações precisas sobre como agir. Isso resulta em maior segurança para a equipe, reduz a variação nas ações realizadas, facilita a adoção de novas tecnologias e promove o uso eficiente dos recursos. Além disso, essas abordagens permitem o monitoramento dos indicadores de processos e resultados, contribuindo para a continuidade dos serviços e a avaliação da qualidade e segurança do atendimento oferecido^(43)^.

O Estudo 10 apresentou um método de “validação clínica de face” com profissionais especializados em prevenção de quedas. Isso levanta questionamentos sobre o significado do termo “clínica”, uma vez que pode remeter à vivência clínica dos profissionais para validar a partir de suas experiências ou ser confundido com a validação clínica propriamente esperada para este estudo. Apesar dessa dicotomia, o estudo foi incluído por empregar o termo e possibilitar um maior esclarecimento e ampliação do método. Nesse contexto, entende-se que alternativas que incluem novos modelos de validação clínica contribuem para superar limitações, permitindo o aperfeiçoamento dos sistemas de linguagem de enfermagem, reduzindo as discrepâncias e aumentando a habilidade dos enfermeiros em reconhecer adequadamente o fenômeno de enfermagem apresentado por um indivíduo^(44)^.

Repara-se que nas três produções iniciais, poucos eram o número de pacientes na amostra, o que não foi justificado teoricamente pelos autores. Presume-se que o tipo de amostra mais utilizada, a não probabilística por conveniência, foi adotada sem critério exposto. Por mais que estes e alguns demais estudos encontrados não adotaram algum método estatístico, a questão amostral é fundamental e precisa ser vista a ponto de demonstrar sua significância e credibilidade do subconjunto.

Espera-se que com o maior número da amostra, maior vai ser a diversificação do julgamento clínico do enfermeiro e consequentemente maior impacto para as práticas assistenciais. Com isso, o que se espera, é que estudos rápidos de serem feitos são mais usuais, podendo dar suporte à prática clínica, mas que necessitam de algum resultado científico que promova validade na tomada de decisão^(45)^. Independente do tamanho da amostra, os critérios adotados precisam ser mais claros, limitações metodológicas e pessoais devem ser apontadas, já que a cronologia acadêmica permite um recrutamento e análise de dados confortáveis.

Os estudos atenderam as recomendações no que diz respeito à validação clínica ser realizada utilizando-se as fases do processo de enfermagem, ou seja, em sua maioria, adotaram instrumentos capazes de atender as fases deste processo, como instrumentos ou fichas de avaliação e exame físico, e o próprio subconjunto validado. Conforme descrito por autoras, a validação do subconjunto terminológico deve apresentar rigor metodológico, seguindo um método estruturado, com base nas cinco etapas do PE, e um padrão conceitual que possibilite embasar essa elaboração^(2)^.

O PE é compreendido como uma ferramenta metodológica que exige competências cognitivas, técnicas e de interação interpessoal. Deve ser desenvolvido e implementado conforme as necessidades do indivíduo, da família ou da comunidade que requer atenção profissional, com ênfase na resolução intencional de problemas^(46)^. Evidentemente, entende-se que a identidade primária da enfermagem reside no cuidado, implementado por meio do PE, que orienta o fazer e o pensar, possibilitando a documentação da prática profissional^(47)^.

Por conseguinte, torna-se plenamente rico a realização da validação clínica com as etapas do PE, fortalecendo uma ciência e valorizando seus profissionais. O PE é o método que orienta o pensamento crítico e o julgamento clínico, necessário com o intuito de estruturar a administração dos cuidados de enfermagem, registrar o atendimento e destacar as atividades desempenhadas por essa equipe em serviços que, muitas vezes, passam despercebidos^(48)^.

A árvore de similitude evidencia a importância da validação clínica originada das produções acadêmicas e reflete o impacto de posicionar a ciência da enfermagem em um padrão aplicável à realidade. Assim, a etapa de validação clínica torna-se essencial para fortalecer a aplicação da CIPE^®^ por enfermeiros assistenciais, promovendo a integração entre os saberes gerados na academia e a prática clínica^(49)^. Contudo, neste contexto, surge o uso do termo “aplicabilidade clínica” nos Estudos 9, 14 e 15, como um eixo que rotula os estudos de validação clínica, inclusive substituindo-o em algumas conceituações de tipo de estudo^(49)^. Essa adoção pode ter sido elaborada com o objetivo de enfatizar a importância de transferir para a prática uma tecnologia do cuidado. A ciência fundamentada em evidências se torna cada vez mais relevante nesse cenário dinâmico e desafiador, onde os resultados das pesquisas são essenciais para questionar, respaldar e transformar as abordagens de ensino, atendimento e gestão de processos^(50)^.

Nota-se da mesma forma que a decisão de comprovar que o enunciado é aplicável, quando este, é aplicado ao menos uma vez, em determinado momento do ato de cuidar, se faça presente, o que justifica sua aplicabilidade clínica^(49)^. Outro modo de validação foi a concordância entre enfermeiros especialistas testada mediante a obtenção de coeficientes sobre confiabilidade interobservadora^(39)^. Independente da forma como se nomeia o estudo, é relevante a necessidade de maior esclarecimento, já que tais estudos sempre foram classificados como de validação, mesmo não tendo sido utilizados índices estatísticos.

### Limitações do estudo

O estudo apresenta algumas limitações. Primeiramente, o grande número inicial de produções foi consideravelmente reduzido, evidenciando apenas o Brasil como produtor de estudos de aplicabilidade clínica dos subconjuntos, o que não pode ser generalizado, já que há uma necessidade de maior aprofundamento no uso dos sistemas de linguagem e métodos utilizados em diferentes continentes. Em segundo lugar, algumas referências não foram encontradas na revisão, exigindo que os autores buscassem informações e o texto completo das produções diretamente nas páginas eletrônicas dos programas de pós-graduação. Isso pode estar relacionado ao fato de autores internacionais utilizarem termos diferentes de “validação clínica”, empregando outros métodos de pesquisa. Por fim, acredita-se que pela variedade de passos metodológicos ou a insuficiente explicação destes nos estudos, algumas informações não foram encontradas claramente, o que pode trazer fragilidade dos resultados encontrados. Ademais, é preciso que as produções acadêmicas sejam divulgadas por meio de artigos científicos a fim de publicizar resultados aglutinados e de fácil acesso.

### Contribuições para a área de Enfermagem

O principal motivo para buscar o entendimento sobre os estudos de validação é aplicá-los na prática assistencial, gerando um impacto positivo para as demandas de saúde da população. Em outras palavras, é necessário “translacionar” esse conhecimento, que é satisfatoriamente alcançado no campo teórico, mas ainda negligenciado no cotidiano da enfermagem. Trata-se, portanto, de um movimento dinâmico que promove avanços contínuos e requer compreensão para gerar novas e promissoras perspectivas. Com isso, os sistemas de linguagem em enfermagem se fortalecem e revelam sua importância como base do processo de enfermagem e da valorização do conhecimento científico da área.

## CONCLUSÕES

Este estudo identificou características e estudos de validação clínica de subconjuntos terminológicos da CIPE^®^. A produção acadêmica tem demonstrado crescimento, com foco principal na saúde do adulto e idoso. Os resultados evidenciam que o Brasil é o principal país produtor nesse tema, com destaque para a região Nordeste, que adotou o estudo de caso como principal referencial metodológico. As análises dos casos ou as análises estatísticas foram os modos majoritariamente usados, mas que carece de avanços, já que os estudos são incipientes e requerem disponibilidade e maior uniformidade de métodos. Esta questão pode dificultar a discussão acerca da fidedignidade dos achados, haja vista que os mesmos indicadores acabam sendo validados para diversos grupos, por exemplo, mas por fases metodológicas completamente diferentes.
